# Surface-Activated Zirconia Nanotubes with UV-Assisted Mg Deposition: Novel Bioinstructive Implants

**DOI:** 10.3390/jfb17030158

**Published:** 2026-03-23

**Authors:** Swathi N. V. Raghu, Yomna Badran, Shanmugapriya Periyannan, Manuela S. Killian

**Affiliations:** Chemistry and Structure of novel Materials (CSnM), University of Siegen, 57068 Siegen, Germany; yomna.badran@student.uni-siegen.de (Y.B.); manuela.killian@uni-siegen.de (M.S.K.)

**Keywords:** magnesium (Mg) decoration, zirconia nanotubes, electrochemical deposition, osseointegration, biomineralization, corrosion resistance

## Abstract

Modern bioimplants increasingly depend on surface-engineered functionality to elicit adaptive biological responses. One promising strategy involves the electrodeposition of bioresponsive elements such as magnesium (Mg), which plays a critical role in osseointegration. In this study, we present a novel approach for modifying anodized zirconia nanotubes (ZrNTs) via Mg decoration using electrochemical deposition. A controlled pulsed cathodic linear sweep protocol was employed to control Mg deposition behaviour, enabling reduced clustering and improved spatial distribution. Notably, ultraviolet (UV) irradiation was found to influence Mg adsorption dynamics, revealing a distinct pattern of interaction. Comprehensive surface characterization was conducted to assess nanotube morphology, Mg adherence, and distribution. These modified surfaces were subsequently evaluated for their potential in further functionalization, targeting surface chemistries conducive to biomaterial viability. The biomineralization capacity of Mg-decorated ZrNTs was systematically investigated using electrochemical impedance spectroscopy (EIS) and Tafel analysis, demonstrating enhanced apatite formation and improved corrosion resistance. This work establishes Mg decoration of ZrNTs as a viable route for developing bioactive, corrosion-resistant implant surfaces.

## 1. Introduction

Orthopedic implants have evolved from passive structural supports to dynamic biointerfaces that actively engage with surrounding tissues. This paradigm shift reflects a growing emphasis on surface functionality, rather than a particularsurface modification in the form of nanoscale architecture, further augmented by the -chemical composition. These surface dependencies act as key determinants of osseointegration, inflammation modulation, and long-term implant viability. Among the materials explored for such applications, zirconia (ZrO_2_) has gained prominence due to its superior mechanical strength, corrosion resistance, and biocompatibility, offering a compelling alternative to titanium-based systems that may pose aesthetic or immunological concerns [1]. Recent advances in electrochemical anodization have enabled the fabrication of zirconia nanotubes (ZrNTs), which exhibit high surface area, tunable porosity, and enhanced interaction with the extracellular matrix (ECM). These nanostructures are increasingly being investigated for their potential in drug delivery, protein immobilization, and bioactive surface engineering [2]. Importantly, unlike bulk polycrystalline zirconia, anodized ZrNTs do not extensively suffer from low-temperature degradation (LTD), a phase transformation issue that compromises mechanical integrity over time. This stability arises from their amorphous or nanocrystalline nature, as reported in recent works [3,4,5,6]. Their phase transformation has reportedly maintained structural integrity under physiological conditions [7,8]. Furthermore, annealing strategies, especially at low temperatures of <350 °C, primarily remove electrolyte-derived residues such as fluorides without inducing LTD-related degradation-associated phase transition. While this structural resilience makes ZrNTs attractive for long-term implantation, their inherent bioinertness limits direct cellular interaction and osteogenic stimulation. To overcome this, surface functionalization strategies have focused on integrating bioactive elements that can endow the material with biological responsiveness without compromising its mechanical integrity.

Magnesium (Mg), a trace element that is naturally abundant in bone tissue, has emerged as a promising candidate for biofunctional surface engineering. Its biological relevance is multifaceted; Mg plays a central role in bone metabolism, stimulates osteoblast activity, and modulates inflammatory responses [9]. Unlike inert coatings, Mg-based modifications offer dynamic interactions with the surrounding tissue, promoting osteoconduction and accelerating early-stage bone healing [10]. Yet, its application in implant coatings is not without controversy. While Mg ions can enhance hydroxyapatite (HAp) nucleation, they simultaneously inhibit lateral crystal growth due to their high hydration energy, often resulting in spherical, amorphous phases rather than continuous mineral layers [11,12]. This dual behaviour has prompted ongoing investigation. While some studies highlight magnesium’s ability to enhance localized bioactivity and protein interactions, others note its tendency toward rapid degradation, which may subtly influence long-term mineral stability. Rather than a drawback, this variability is often viewed as a tunable feature, one that can be optimized through surface engineering and treatment conditions [13,14].

To harness magnesium’s biofunctional potential while mitigating its limitations, we explored electrochemical deposition techniques that offer precise control over surface modification. Prior studies, such as Yoshioka et al.’s work on electrochemically assisted sol–gel deposition, demonstrated how electrochemical stimuli can influence the formation and distribution of bioactive coatings [15,16]. Although their approach focused on gel-based systems, it prompted us to consider whether similar electrochemical principles, specifically pulsed LSV (pLSV), could be adapted for direct magnesium incorporation onto anodized zirconia nanotubes. This method, involving forward and reverse sweep pulses, will allow fine-tuning of nucleation density, particle morphology, and spatial distribution, offering a promising route to engineer surfaces with enhanced biological functionality. Building on this conceptual foundation, our study investigates Mg decoration of ZrNTs using pLSV, with and without UV assistance, to evaluate how deposition parameters influence surface morphology and bioactivity [15]. Moreover, external stimuli like ultraviolet (UV) irradiation have been shown to influence electrochemical kinetics and surface charge dynamics, offering an additional layer of control over Mg deposition behaviour. In aqueous electrolytes, UV does not activate the ZrNTs directly, but it can modify the electrolyte environment by generating reactive oxygen species, accelerating the breakdown of organic contaminants, and transiently altering wettability, surface charge, adsorption behaviour, and capillary imbibition. These UV-induced shifts in interfacial conditions can enhance Mg^2+^ interaction with the nanotube surface. Previous reports have suggested that UV irradiation increases surface hydroxylation, which facilitates Mg^2+^ adsorption and distribution [16,17]. This synergistic effect supports uniform mineral deposition, as previously shown for TiO_2_, and is transferable to ZrO_2_-based systems [18]. However, the reproducibility and mechanistic understanding of UV-assisted deposition remain underexplored, and its integration into bioimplant design is still in its infancy.

In this study, we investigate a strategy for decorating anodized ZrNTs with Mg using pLSV during fabrication, with and without UV assistance. By leveraging the high surface area and chemical stability of ZrNTs, we aim to create bioactive interfaces with the potential to support protein immobilization, modulate enzymatic activity, and improve corrosion resistance. The influence of Mg decoration on surface morphology and hydroxyapatite formation is examined, with a focus on understanding how electrochemical parameters and UV exposure shape the biofunctional landscape of the modified implant surface.

## 2. Materials and Methods

### 2.1. Fabrication of ZrO_2_ Nanotubes (ZrNTs)

High-purity zirconium foils (99.6%, 0.127 mm thickness; Thermo Fisher GmbH, Waltham, MA, USA) were sectioned into 1.5 × 1.5 cm^2^ specimens and subjected to sequential ultrasonic cleaning in deionized water, ethanol, and acetone for 15 min per solvent to eliminate surface contaminants. Electrochemical anodization was conducted in a custom O-ring cell configuration, employing a platinum foil as the counter electrode and the zirconium specimen as the working electrode. The anodization electrolyte comprised 2 wt% H_2_O_2_ wt% NH_4_F (Carl Roth, Karlsruhe, Germany, 98%), and 30 vol% formamide (Acros Organics, Geel, Belgium, 99.5%) in glycerin (Sigma-Aldrich, St. Louis, MI, USA, 99%). The anodization process was performed via a high-voltage potentiostat (Jaissle IMP 88-200 PC, Münster, Germany), where direct current (DC) voltage was ramped to 90 V over 5 min and maintained for 60 min to facilitate the formation of zirconia nanotubular structures. Following anodization, the samples were thoroughly rinsed with deionized water and ethanol, immersed in ethanol for 120 min to enhance pore stabilization, and subsequently dried under a nitrogen stream. A final thermal treatment was performed by annealing the samples using a controlled heating profile, in which the temperature was ramped up at 5 °C/min, then held at 300 °C for 60 min under ambient atmospheric conditions to induce crystallinity and improve structural integrity.

### 2.2. Magnesium Decoration via Electrochemical Deposition

Electrochemical deposition of magnesium onto ZrO_2_ nanotubes (ZrNTs) was performed using a three-electrode setup comprising ZrNTs as the working electrode, platinum as the counter electrode, and Ag/AgCl (vs. 3M KCl, Metrohm, Herisau, Switzerland) as the reference electrode. The electrolyte consisted of 0.5 M KOH, 0.5 M Na_2_SO_4_, and 100 µM Mg(NO_3_)_2_·6H_2_O. Deposition was carried out under ambient conditions, both in the presence and absence of UV illumination (680 mW–365 nm), using a Biologic SP-200 potentiostat (Biologic Science Instrument, Seyssinet-Pariset, France). To activate Mg nucleation sites, the nanotube surface was conditioned through a 1 min chronoamperometric pulse at −1.200 V, which removes loosely bound species and induces mild electrochemical reduction. Under UV illumination, additional photochemical cleaning of the electrolyte–surface interface occurs, including the breakdown of organic residues and transient shifts in wettability and surface charge. These combined effects further promote Mg nucleation by increasing the density and accessibility of reactive adsorption sites. This enhances hydroxide mobility and exposes defect states to facilitate Mg binding. This was followed by 50 cycles of linear sweep voltammetry (LSV), designed to control growth dynamics: the nucleation phase (−1.450 V to −1.600 V, @15 mV/s) enhanced localized Mg reduction and enabled its adherence to ZrO_2_ nanotubes, while the refinement phase (−0.500 V to −0.300 V, @5 mV/s) facilitated surface diffusion and stabilization of Mg. The latter interrupted the excess growth occurring during the nucleation phase, thereby discouraging cluster formation. This dual-step protocol ensured consistent and conformal Mg decoration, which is critical for optimizing surface functionality.

During cathodic polarization, water reduction will occur at the electrode surface according to

$$2H_{2}O+2e^{-}\to H_{2}+2OH^{-}$$

This reaction will then induce localized alkalization at the electrode–electrolyte interface, generating a high concentration of OH^−^ ions in the near-surface region. This will further promote interfacial precipitation of magnesium hydroxide according to

$$Mg^{2+}+2OH^{-}\to Mg(OH{)}_{2}(s)$$

Mg deposition via this pLSV technique does not occur as metallic Mg electrodeposition, which is thermodynamically unfavourable in aqueous alkaline media. The pLSV steps involved are expected to assist in allowing electrochemically induced nucleation and lead to the growth of Mg(OH)_2_ species at the ZrNT interface. Partial dehydration or interfacial bonding may also yield Mg-O-Zr linkages and MgO-like domains upon stabilization. Thus, throughout this manuscript, the term “Mg deposition” refers to the electrochemically assisted interfacial attachment of Mg(OH)_2_/MgO-type species onto the ZrO_2_ nanotube surface, rather than metallic Mg electrodeposition.

Further, UV-assisted deposition was performed using a 365 nm light source (with nominal power: 680 mW) positioned at a fixed distance of approximately 7 cm (diagonally) from the sample surface. The guiding probe was fixed such that constant geometry was maintained, and the diverged emission fully covered the circular anodized area fitted to a customized Teflon electrochemical cell. The same cell configuration and optical alignment were maintained for all light-assisted experiments to ensure uniform and reproducible irradiation conditions. No noticeable bulk temperature rise, or any other visible effects, were observed during irradiation, suggesting that the observed differences in sample properties arise primarily as a result of UV-induced interfacial processes rather than thermal effects.

### 2.3. Biofunctional Assessment of Mg-Decorated ZrO_2_ Nanotubes

To evaluate bioactivity, samples were incubated in simulated body fluid (SBF, pH 7.4) at 37 °C for 7 days to promote and assess their biomineralization potential. Mineral deposits containing calcium (Ca) and phosphate (P) contribute to the formation of hydroxyapatite (HAp), a key indicator of the osteoconductive properties of implant materials. Surface mineralization was characterized using scanning electron microscopy (SEM) and X-ray diffraction (XRD), which confirmed both the presence and crystallinity of the deposited mineral phases.

Corrosion resistance and biostability were further examined through hydrogen evolution analysis, which serves as a proxy for Mg degradation in aqueous environments. Gas chromatography (SRI 8610C, thermal conductivity detector) was used to quantify the evolution of the byproducts of Mg degradation, i.e., hydrogen gas (H_2_), at specific time intervals, providing insights into the kinetics of surface dissolution of the Mg-ZrNTs and their implications for implant longevity.

To assess the suitability of Mg-ZrNT surfaces for biofunctionalization, we probed protein adsorption and enzymatic compatibility using horseradish peroxidase (HRP) as a model enzyme. Surfaces were chemically activated with 25 mmol/L 1,1′-carbonyldiimidazole (CDI) in chloroform, enabling covalent immobilization of HRP in phosphate-buffered saline (PBS, pH 6.4). The enzymatic activity of immobilized HRP was evaluated using a colorimetric ABTS assay, with absorbance monitored at 747 nm via UV–Vis spectroscopy. This functionalization strategy simulates biointerface interactions and confirms the retention of catalytic activity post-immobilization, thereby validating the surface chemistry of Mg-ZrNTs for enzyme compatibility.

### 2.4. Electrochemical and Surface Characterization

Electrochemical impedance spectroscopy (EIS) and Tafel analysis were conducted in PBS (pH 7.4) at 37 °C using the same three-electrode configuration. Impedance spectra were recorded over 300 kHz to 0.1 mHz with a 10 mV AC amplitude and analyzed using EC-lab software.

Morphological and compositional analysis were performed using scanning electron microscopy (ESEM FEI Quanta 250, Eindhoven, The Netherlands), X-ray photoelectron spectroscopy (Evans Analytical Group LLC XPS SSX-100 (XPS), S-probe, Santa Clara, CA, USA), and energy-dispersive X-ray spectroscopy (SEM-EDX). All XPS measurements were carried out at a normal angle with monochromatized Al Kα radiation and all the high resolution spectra were calibrated using C 1s as reference peak set at 284.8 eV. All XPS related spectra were plotted and fitted using CasaXPS software (Casa software Ltd., Teignmouth, UK); data were calibrated and normalized. Magnesium presence was confirmed via ion fragment detection using time-of-flight secondary ion mass spectrometry (ToF-SIMS4, ION-TOF GmbH, Münster, Germany). Wettability changes were assessed through water contact angle measurementsOssila goniometer Sheffield, UK) following surface functionalization with octadecylphosphonic acid (ODPA) and 8-hydroxyquinoline (8HQ), used to probe Mg incorporation indirectly as a first measure. For this, contact angle measurements were performed under ambient laboratory conditions (20–23 °C, relative humidity 15–25%) using 10 µL DI water dispensed for each droplet measurement. For every sample, five independent replicates were analyzed, and on each replicate, the contact angle was measured at three different surface positions. The reported values represent the mean of these measurements.

## 3. Results and Discussion

**Figure 1:**
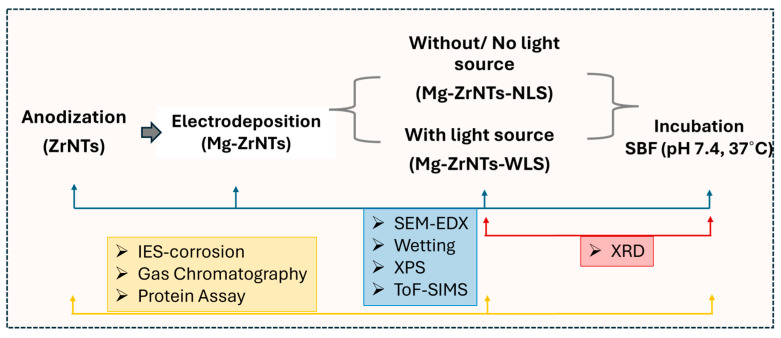
Schematic representation of the experimental overview.

To evaluate the multifunctional potential of Mg-modified zirconia nanotubes, we first synthesized well-defined nanotubular architectures and introduced magnesium to the nanostructures via electrochemical deposition (with and without UV treatment). Initial characterization was performed using SEM-EDX to confirm morphological integrity and phase composition. These substrates were then incubated in simulated body fluid (SBF) to assess bioactive deposition and subsequently analyzed via XRD to identify the phase composition of the resulting biomineral deposits, followed by surface wettability measurements and XPS and ToF-SIMS analysis to verify magnesium presence and distribution. A schematic representing the experimental and analysis flow is shown in Figure 1.

With surface chemistry established, we proceeded with electrochemical impedance spectroscopy (EIS), corrosion testing, and gas chromatography to probe implant-relevant behaviour. Finally, protein–substrate compatibility was assessed via UV–Vis spectroscopy using horseradish peroxidase (HRP) to determine biofunctionality across the three engineered substrate types.

### 3.1. Morphological Studies

#### 3.1.1. SEM and EDX

**Figure 2:**
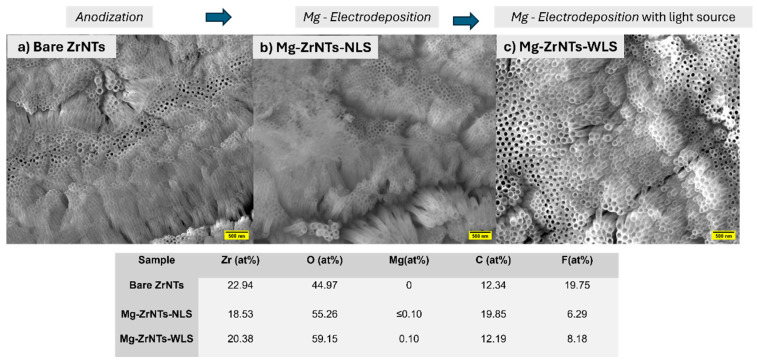
SEM (**top**)—(**a**) Bare ZrNTs, (**b**) Mg-ZrNTs-NLS, (**c**) Mg-ZrNTs-WLS, where NLS implies no light source and WLS is with light source, and EDX (**bottom**) analysis of ZrNTs samples under different treatment conditions.

In Figure 2, the top panel presents scanning electron microscopy (SEM) images of three sample surfaces: zirconia nanotubes (ZrNTs) formed via anodization of zirconium and electrodeposited Mg-ZrNTs (with/without UV light sources). These specimens will be denoted as Bare ZrNTs, Mg-ZrNTs-NLS (NLS—no light source), and Mg-ZrNTs-WLS (WLS—with light source) throughout this manuscript. All samples exhibit densely packed nanotube arrays with open tube tops. The average nanotube diameter is approximately 90 ± 10 nm. The bare ZrNTs show uniform surface morphology and ordered arrays that are similar to Mg-ZrNTs-NLS, with no notably distinguishing features. However, the Mg-ZrNTs-WLS samples display a significantly higher contrast following UV-assisted magnesium electrodeposition. The micrograph appears brighter, likely due to surface charging effects during imaging, which can be attributed to additional material on the nanotubular surfaces [19].

The bottom panel shows energy-dispersive X-ray spectroscopy (EDX) data for the same samples, summarizing the atomic percentages of key elements. Magnesium is absent in bare ZrNTs but detected in both electrodeposited samples [6,20,21,22,23]. The Mg-decorated samples show Mg signals below 0.1 at%, which are detectable but too close to the instrumental sensitivity limit to be taken at face value as a quantitative measure of loading. At this stage, the presence of Mg is therefore interpreted qualitatively rather than used to infer absolute deposition efficiency, and EDS data indicate only that an additional surface-associated species is present; the nature of this modification becomes clearer in the subsequent analyses presented later in the study [19]. Both Mg-ZrNTs samples exhibit an increase in oxygen content. Although this is compatible with the formation of MgO or Mg(OH)_2_ species on the surface [20], the magnitude of the O increase (10–15%) exceeds what would be expected from hydroxylation alone. This indicates that multiple mechanisms likely contribute to the oxygen-rich surface chemistry. Plausible contributors include Mg-Zr substitution at defect-associated oxygen vacancies, formation of Mg(OH)_2_ domains, increased water retention within the nanotube layer, and the higher effective surface area created by the deposition process. UV-assisted samples further support this broader interpretation. In aqueous electrolyte, UV exposure does not directly activate ZrO_2_ but modifies the interface by photochemically removing organic contaminants and generating a transiently more hydroxylated, more hydrophilic surface. The formation of Zr-OH_x_ species, together with UV-induced shifts in wettability and surface charge, increases the accessibility of reactive adsorption sites. These effects can facilitate Mg nucleation and contribute to the observed oxygen enrichment, but they cannot fully account for the total O increase on stoichiometric grounds alone. UV irradiation influences the adsorption kinetics of Mg^2+^ by accelerating the formation of surface hydroxyl groups and increasing the transient negative surface charge of the ZrNTs. These UV-induced changes reduce the energy barrier for Mg^2+^ adsorption, increase the density of available coordination sites, and enhance ion transport within the wetted nanotube layer. As a result, Mg^2+^ ions adsorb more rapidly and uniformly onto the nanotube walls, which in turn affects the nucleation rate and early-stage growth kinetics during electrodeposition. Variations in Zr, O, C, and F content across treatments are also observed. The less-relevant C and F signals originate from residues associated with the anodization electrolyte [6,21,22]. Samples subjected to electrodeposition show a reduction in fluorine intensity, consistent with partial displacement or coverage of fluoride-containing surface species during Mg decoration. This depletion likely results from competitive ion exchange and surface restructuring during the second electrochemical step. KOH introduces hydroxide ions that displace surface-bound fluoride, while Mg^2+^ deposition alters local coordination environments, reducing fluoride retention on the ZrO_2_ nanotube surface [24].

**Figure 3:**
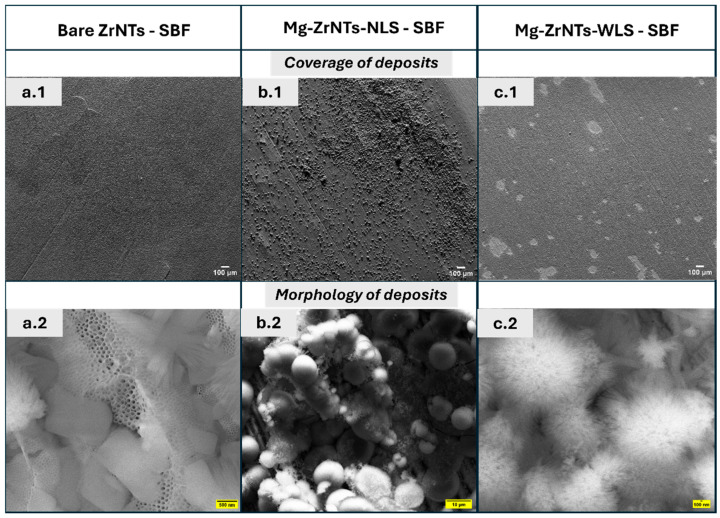
SEM images (**top**) as synthesized substrates: (**a.1**) Bare ZrNTs, (**b.1**) Mg-ZrNTs-NLS, and (**c.1**) Mg-ZrNTs-WLS, and (**bottom**) after 7-day incubation in simulated body fluid (SBF): (**a.2**) Bare ZrNTs, (**b.2**) Mg-ZrNTs-NLS, and (**c.2**) Mg-ZrNTs-WLS.

After preliminary characterization, these samples were immersed in SBF for 7 days under static conditions at 37 °C to assess surface mineralization ability. In Figure 3, the previously opened tube-tops exhibit a well-defined, noticeable coverage of surface deposits, strongly indicative of mineralization. The bare ZrNTs show some discontinuity in between what appears to be uniform coverage as seen in the top half of the figure. This contrasts with Mg-ZrNTs-NLS, which display moderate deposition with granular morphology. Interestingly, Mg-ZrNTs-WLS exhibit extensive surface coverage, with dense and heterogeneous deposit structures.

In the bottom half of Figure 3, clear differences in the morphology of the deposits are evident. The bare ZrNTs display some open tube-tops along with cuboidal fragments partially covering the surface. In the Mg-ZrNTs-NLS sample, clusters of spherical deposits are observed, surrounded by a mix of smooth and fuzzy particles. The diameter of these spheres is estimated to average between 1 and 10 µm. The most striking features appear on the Mg-ZrNTs-WLS specimen, where the deposits resemble blooms radiating from a central sphere. The uniform distribution and dense coverage of mineral deposits, presumably apatite on the Mg-decorated samples, may be attributed to the homogeneous dispersion of magnesium across the surface [25]. The uniformity is likely supported by UV irradiation, which transiently increases the hydrophilicity of the zirconia nanotube surface by promoting interfacial hydroxylation and removing weakly bound organic species [24]. In Figure S1, the comparison between the non-Mg electrodeposited ZrNTs and the UV-treated bare ZrNTs, shown by SEM together with the corresponding chemical analysis, further highlights the effect of UV activation and supports the hypothesis that the presence of Mg influences the resulting CaP morphology. The resulting increase in accessible, polar surface sites enables more consistent magnesium incorporation, which then favours localized supersaturation and nucleation of calcium phosphate phases during SBF incubation [26]. Magnesium ions play a critical role in modulating hydroxyapatite (HAp) crystallization by stabilizing the amorphous calcium phosphate (ACP) precursor phase. Through partial substitution of Ca^2+^ in the calcium phosphate lattice, Mg^2+^ disrupts the orderly progression of crystal growth, thereby inhibiting the formation of large, well-defined HAp crystals [26]. This interference promotes the retention of nanoscale, fine-grained apatite particles, as evident in the morphological features observed in Figure 3. This type of regulation of the mineral phase development is consistent with magnesium’s known influence on nucleation kinetics and crystal maturation pathways [27,28]. Collectively, these images suggest pronounced treatment-dependent variation in bioactivity and nucleation behaviour and are consistent in indicating the presence of Ca/P deposition, like the constituents of ACP, and growth during the soaking duration. Such morphologies align with Kokubo’s SBF study, which reported that apatite needle-shaped structures were observed on different bioactive materials upon soaking in SBF [29].

#### 3.1.2. XRD Analysis—SBF Incubation

**Figure 4:**
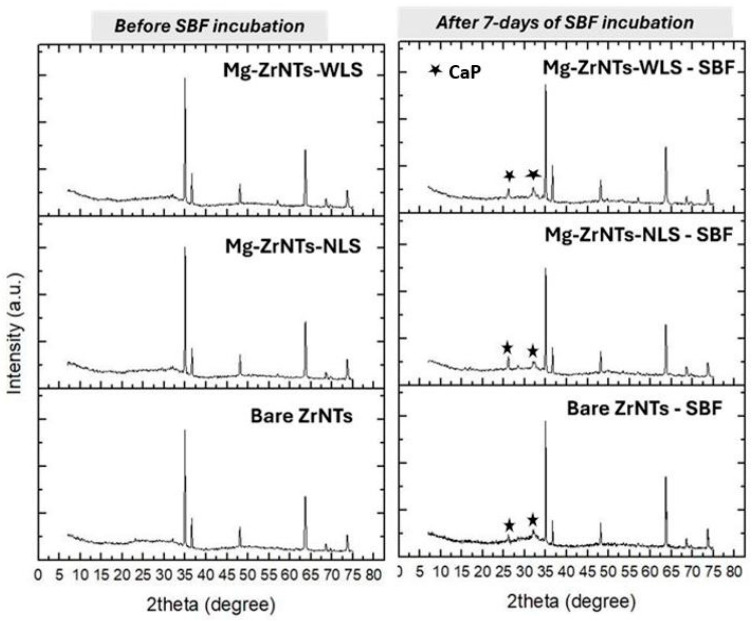
XRD analysis of Bare ZrNTs, Mg-ZrNTs-NLS, and Mg-ZrNTs-WLS samples before (**left**) and after (**right**) SBF incubation.

To complement the morphological insights gained from SEM, X-ray diffraction (XRD) analysis was performed on Bare ZrNTs, Mg-ZrNTs-NLS, and Mg-ZrNTs-WLS samples both before and after SBF incubation, and the results are shown in Figure 4. This technique was used to identify the crystalline phases present and to determine whether the surface deposits correspond to bioactive mineral phases.

In the left panel, before SBF incubation, all three samples, bare-Zr_2_ Mg-ZrNTs-NLS, and Mg-ZrNTs-WLS, exhibit the same set of diffraction peaks at 2θ values of 34.94°, 36.6°, 48.1°, 63.64°, 68.5°, and 73.6°. These peaks can be assigned to the Zr metal substrate, as discussed in the literature [3,6,30,31,32]. Such substrate-related peaks are commonly observed beneath anodized ZrO_2_ nanotubular layers, particularly when the oxide layer is thin or only partially crystalline. In these cases, X-rays can penetrate the nanotube structure and diffract from the metallic zirconium lattice [23,32]. Following immersion in simulated body fluid (SBF), new diffraction peaks emerged at 2θ = 26.0° and 32.07°, corresponding to the (002) and (112) planes of calcium phosphate (CaP), respectively [33,34,35]. These features indicate early-stage calcium phosphate deposition on the surface. The characteristic triplet band of Hap, (211)/(112)/(300), was not distinctly resolved, instead appearing as a broad envelope, likely due to the short soaking duration and incomplete crystallization [36,37]. Importantly, the underlying ZrO_2_ nanotube (ZrNT) signals remained visible at their original positions, confirming that the nanotubular architecture was preserved despite surface mineralization. It is noteworthy that bulk zirconia is intrinsically bioinert and does not spontaneously nucleate Ca-P phases. The early Ca-P deposition observed here arises from the synergistic combination of the nanotubular morphology, which enhances fluid imbibition and ion exchange, and the Mg-induced increase in surface reactivity. This cooperative effect enables the formation of Ca-P precursor phases that would not typically occur on unmodified zirconia surfaces.

### 3.2. Surface Analysis

To systematically verify magnesium incorporation on the ZrNT surface, we employed a tiered characterization strategy based on depth sensitivity. Each technique interrogates a distinct interfacial zone: water contact angle measurements probe the outermost molecular layer, reflecting changes in surface chemistry and wettability; ToF-SIMS accesses the near-surface region with nanometer-scale resolution, enabling spatial mapping of elemental fragments; and X-ray photoelectron spectroscopy (XPS) provides compositional insights from the top ~5–10 nm, confirming chemical states and elemental presence [38]. This progressive approach from surface reactivity to subsurface composition offers convergent evidence for successful magnesium deposition.

#### 3.2.1. Water Contact Angle

**Figure 5:**
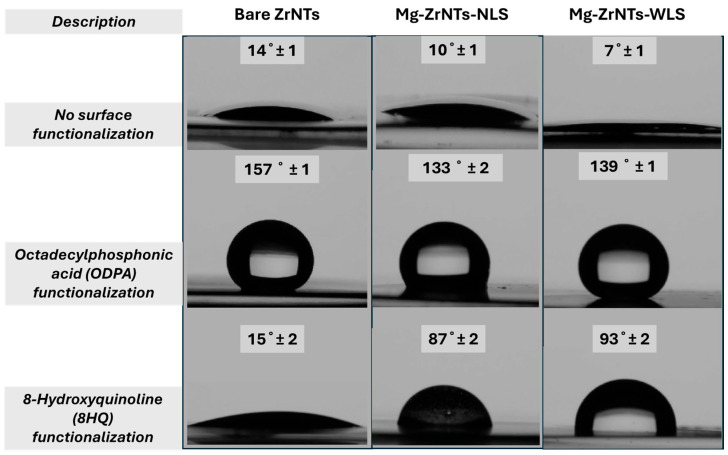
Water Contact Angle (WCA) measurements on substrates (**top**), unmodified—as prepared, (**middle**) ODPA—modified, and (**bottom**) 8HQ—modified.

To assess whether Mg was successfully deposited on the ZrNT surface, we employed a two-step indirect probing strategy. First, both bare and Mg-decorated zirconia nanotubes were functionalized with octadecylphosphonic acid (ODPA), and a second set was functionalized with 8-hydroxyquinoline (8HQ). In Figure 5, the digital photographs show the water contact angle formed on each of the modified substrates. The unmodified substrates show a hydrophilic response, a behaviour that was expected due to the capillary action of the nanotubes [39]. The extreme wetting is also attributed to the presence of surface hydroxyl groups on metal-oxide substrates. In the panel depicting ODPA-functionalization, a superhydrophobic response is clearly noted for bare ZrNTs, and this observation aligns with trends previously reported in the literature, indicating consistent surface behaviour across similar systems [40,41]. However, on the Mg-decorated substrates, a reduction in water contact angle is observed, though the surfaces still exhibit a hydrophobic response. The shift in wettability likely reflects changes in surface reactivity introduced during electrodeposition, together with the presence of hydrophilic magnesium-containing species on the nanotube walls. Magnesium deposited under these conditions is plausibly stabilized as Mg(OH)_2_ or MgO, both of which increase surface polarity and contribute to the observed hydrophilicity. The Mg-ZrNTs-WLS sample shows the strongest wettability shift, consistent with more extensive magnesium incorporation under UV-assisted conditions. In this context, UV illumination is understood to enhance hydrophilicity primarily by cleaning the interface, removing weakly bound organic residues and promoting transient interfacial hydroxylation in the aqueous electrolyte, thereby increasing the density of accessible -OH groups without invoking photocatalytic pathways. These combined effects provide a more receptive surface for magnesium attachment and help explain the enhanced wettability [42]. To confirm that this disruption was due to the presence of magnesium, we subsequently treated the surfaces with 8-hydroxyquinoline (8HQ), a chelating agent known to selectively bind Mg^2+^. Interestingly, in the panel depicting 8HQ functionalization, the trend is reversed; all substrates displayed hydrophilic behaviour, with bare ZrNTs showing reactivity comparable to unmodified controls. The Mg-doped ZrNTs, in particular, exhibit contact angles at the higher end of the hydrophilic range (15–90°) corresponding to the availability or continuity of Zr-OH binding sites [43]. This behaviour may indicate reduced surface hydrophilicity due to the formation of an insoluble, surface-anchored magnesium–quinoline (Mg-Q_2_) complex. The enhanced binding affinity of 8-hydroxyquinoline (8HQ) toward Mg-modified surfaces, relative to bare ZrNTs, reflects differences in the chemical state and coordination environment of surface cations [44,45]. As a bidentate ligand, 8HQ selectively chelates Mg^2+^ via its quinoline nitrogen and hydroxyl oxygen atoms, forming stable tetradentate metal complexes [46]. Another key observation is the contact angle measured for Mg-ZrNTs-WLS, which exhibits the highest value (93 ± 2°), indicating greater 8HQ binding relative to the non-illuminated sample. This supports the indication of enhanced Mg deposition. UV-assisted electrodeposition can promote greater Mg uptake by improving the interfacial conditions of the ZrNT surface, primarily through the removal of weakly bound organic residues and the transient formation of additional surface hydroxyl groups in the aqueous electrolyte rather than by generating oxygen vacancies through photocatalytic pathways [42,47]. Consequently, more surface Mg^2+^ ions become available for chelation by 8HQ, resulting in a higher density of surface-bound complexes, increased hydrophobicity, and elevated contact angles. These findings reinforce the effectiveness of UV-assisted deposition in modulating surface chemistry and confirm its role in promoting uniform and reactive magnesium decoration.

#### 3.2.2. ToF-SIMS Analysis

**Figure 6:**
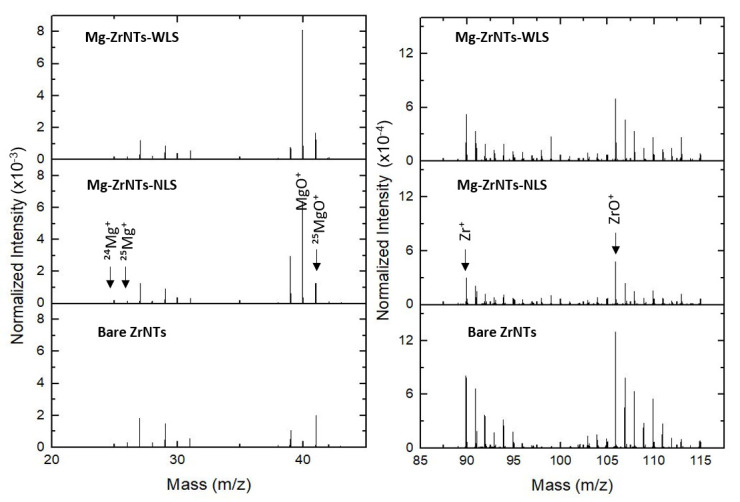
Normalized positive-ion ToF-SIMS spectra of bare and Mg-decorated ZrO_2_ NTs (NLS, WLS): (**left**) *m*/*z* 24–44 region showing Mg isotopes and oxygenated fragments; (**right**) *m*/*z* 80–115 region highlighting zirconia-related species.

**Figure 7:**
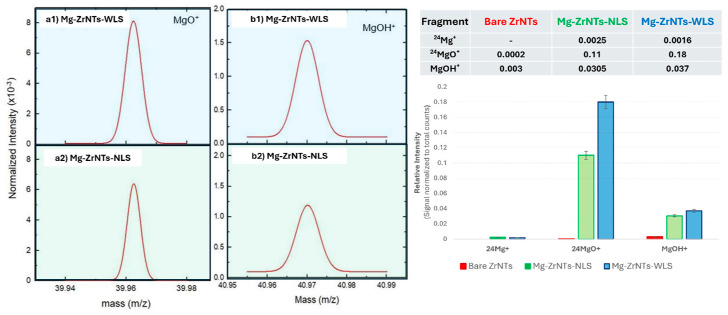
(**Left**) Normalized positive-ion spectra comparing (**a1**,**b1**) WLS and (**a2**,**b2**) NLS samples for two representative fragments (^24^Mg^+^ and MgO^+^) and (**Right**) bar graph and data table showing normalized intensities of key Mg-related fragments on all substrates.

While water contact angle measurements provide a rapid, surface-sensitive indication of magnesium incorporation, they remain indirect. To substantiate these findings and probe the spatial distribution and chemical identity of the deposited species, these results are complemented by time-of-flight secondary ion mass spectrometry (ToF-SIMS). This technique offers high-resolution chemical mapping and depth-sensitive analysis, enabling direct confirmation of magnesium presence across the ZrNTs surface. In Figure 6, ToF-SIMS analysis provides direct evidence of magnesium presence on ZrNTs. In positive-ion mode, characteristic fragments like Mg^+^, MgO^+^, and MgOH^+^ were detected on the Mg-decorated samples but absent in bare ZrNTs, confirming successful deposition. Isotopic resolution enabled identification of ^24^Mg^+^, ^25^Mg^+^, and ^26^Mg^+^ at *m*/*z* 23.96, 24.46, and 25.97, respectively, along with oxygenated species at *m*/*z* 39.98 (MgO^+^), 40.98 (^25^MgO^+^), and 41.95 (^26^MgO^+^). The presence of hydroxylated fragments suggests that magnesium may be stabilized on the surface as oxide or hydroxide species. Furthermore, in the left panel in Figure 6, substrate-based fragments (Zr^+^, ZrO^+^) are presented. Notably, Zr signal intensity decreases progressively from bare to NLS to WLS samples, indicating surface modification by Mg. This suppression may result from Mg masking the Zr surface or altering ionization efficiency. In Figure 7, although the raw Mg signal intensity appeared lower in WLS samples, normalization to total ion counts and substrate signals revealed higher relative Mg content, confirming more effective doping via UV-assisted deposition [48].

A comparative analysis of Mg-decorated ZrNTs prepared with and without UV illumination revealed distinct differences in fragment intensities. NLS samples showed stronger signals for metallic Mg, while WLS samples exhibited enhanced MgO^+^ and MgOH^+^. This shift indicates that UV-assisted deposition promotes oxidation and hydroxylation of surface-bound Mg. Photochemical activation likely enhances local oxygen adsorption, favouring Mg-O and Mg-OH bond formation over metallic clustering [49]. These transformations increase the yield of oxygenated Mg fragments under positive polarity and reduce metallic Mg^+^ signals. These ToF-SIMS results build directly on the wettability trends observed via contact angle measurements, confirming that the changes in surface wetting behaviour are indeed attributable to the presence and chemical state of deposited Mg species.

#### 3.2.3. XPS Analysis

To further investigate the chemical state and surface environment of the deposited magnesium, XPS was employed. This technique provides elemental specificity and oxidation state resolution, enabling direct assessment of Mg bonding configurations and molecular interaction with the ZrNTs and Mg-ZrNTs (NLS/WLS) substrates. Additionally, XPS was also performed on samples post-SBF incubation to investigate Mg-induced CaP formation, an effect previously suggested by morphological changes seen in Figure 3 and Figure 4. This analysis aims to resolve the chemical basis of surface mineralization and validate Mg’s role in promoting bioactive phase development.

**Figure 8:**
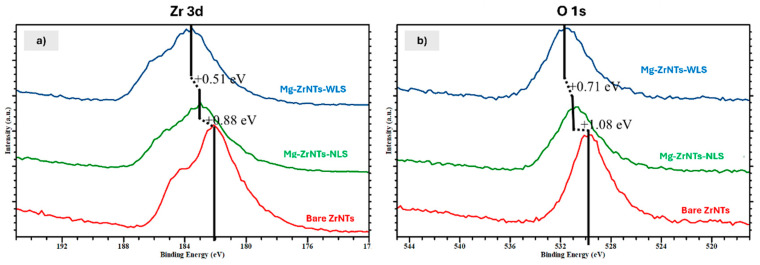
(**Left**) (**a**)—XPS spectra showing the Zr3d and (**Right**) (**b**)—O1s peaks of bare-ZrNTs, Mg-ZrNTs-NLS, and Mg-ZrNTs-WLS.

In the analysis, changes in the chemical environment of Zr and shifts in the O 1s region were examined to assess the impact of Mg deposition. As seen in Figure S2 (in the Supplementary Information), the survey spectra confirm the presence of Zr and O across all samples, consistent with the ZrO_2_ substrate. No additional signals were observed, other than adventitious carbon. For SBF-incubated samples, peaks corresponding to calcium (Ca) and phosphorous (P) emerged, indicating surface interaction with bioactive ions and supporting the hypothesis of Mg-induced mineralization. The narrow scans of Zr 3d and O 1s peaks for ZrO_2_, showes sharp lattice-derived features at 182.1 eV (Zr 3d) and 529.72 eV (O 1s), which can be associated with the stoichiometric Zr^4+^-O^2−^ bonding [50]. After the deposition of Mg under NLS conditions, these peaks show a noteworthy shift (≈0.88 and 1.08 eV) toward higher binding energy, as seen in both panels in Figure 8 and Table 1.

Interestingly, for Mg-ZrNTs-WLS, a further shifting of the binding energy to higher values, ≈0.51 and 0.77 eV, is noted. The observed shift indicates electron withdrawal from Zr-O lattice bonds, consistent with Mg incorporation into the surface lattice and the formation of bridging Zr-O-Mg linkages. The significant shifts in the Zr 3d (≈0.88–1.39 eV) and O 1s (≈1.08–1.85 eV) core-level peaks following Mg deposition are indicative of the formation of a ZrO_2_-Mg interfacial region, accompanied by substantial electronic and chemical restructuring [51,52].

**Figure 9:**
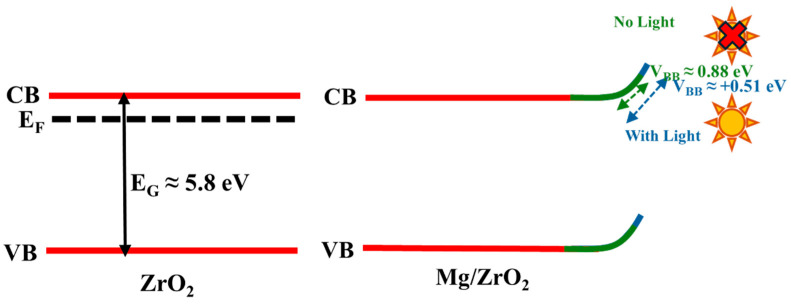
Energy Band diagrams of (**left**) Bare-ZrNTs showing the theoretical bandgap, and (**right**) Mg-ZrNTs-NLS and Mg-ZrNTs-WLS surfaces illustrating experimentally determined valence band bending.

The progressive shift in Zr 3d binding energy from bare to NLS and WLS samples reflects surface electron withdrawal and corresponds to upward band bending, as shown in Figure 9 [53]. The Mg 2p signal corresponding to MgO/Mg(OH_)2,_ which occurs in the range of 49.5–51.5 eV, overlaps significantly with the Zr 4s region, which lies between 50 and 55 eV, making the direct spectral attribution challenging. However, clear differences in the evolution of the Zr 4s region, as shown in Figure S3, before and after the Mg deposition, provide indirect but strong evidence for Mg’s addition to the ZrO_2_ matrix. Notably, the Zr3d shift systematically moves towards higher binding energies for both the NLS and WLS conditions, indicating electron withdrawal and upward band bending upon Mg deposition (Figure 9), but Zr 4s shows a different evolution. While NLS in Zr 4s follows a positive shift, WLS exhibits a slight shift towards lower binding energy. This alternating shift suggests that the shallow Zr 4s level is more sensitive to local screening and polarization effects at the interface as a result of increased hydroxylation under WLS conditions. Therefore, Zr 4s evolution reflects interfacial electronic screening, while Zr 3d indicates the overall electron withdrawal mechanism [54]. Concurrently, a discernible increase in the shoulder intensity of the O 1s spectrum (≈531.5 eV) is observed across the series (bare ZrO_2_ ➝ NLS ➝ WLS), corresponding to hydroxide species and indicating progressive surface hydroxylation. This trend underscores the increase in surface hydroxylation, most notably in the WLS sample, where UV illumination during deposition appears to promote the formation of Mg(OH)_2_-rich domains. This, in turn, facilitates the development of Zr-O-Mg(OH)_2_ interfacial linkages [55]. The associated evolution in electronic structure is corroborated by ToF-SIMS data, which show enhanced Mg^+^ and MgOH^+^ signals for WLS. Furthermore, the formation of a more hydrated, dipole-rich surface in WLS is reflected in an additional core-level shift of ~0.5 eV relative to NLS.

Overall, Mg deposition on ZrNTs leads to the formation of Zr-O-Mg linkages and hydroxyl-rich domains that modify the surface dipole strength. This consequently alters the electrostatic potential and hence the observed shifts in Zr 3d, Zr 4s and O 1s binding energies. Collectively, these findings indicate that chemically elusive and even low-level Mg incorporation can be electronically significant, inducing substantial charge redistribution and hydroxylation across the ZrO_2_ nanotube surface. This modified interfacial chemistry establishes a favourable platform for enhanced reactivity and subsequent hydroxyapatite nucleation, as discussed in subsequent sections.

Previously, in Figure 3, SBF incubation resulted in distinct surface deposits across all substrates. XPS analysis of these samples revealed clear Ca 2p and P 2p signals on all surfaces, confirming Ca-P deposition. Atomic percentage quantification was performed using background-corrected peak areas, scaled by relative sensitivity factors (RSF) and normalized to the total signal. The data is represented in Table 2. Among the three surfaces, Mg-ZrNTs-WLS exhibited the most pronounced attenuation of the Zr signal, consistent with the formation of a more continuous Ca-P/Mg overlayer following immersion.

All samples displayed Ca/P ratios below the stoichiometric value for hydroxyapatite (HAp, Ca/P = 1.65), in line with classical SBF assay results reported by Kokubo [29]. The measured Ca/P ratios were 0.266 (bare-ZrNTs), 0.170 (Mg-ZrNTs-NLS), and 0.222 (Mg-ZrNTs-WLS), indicative of Ca-deficient amorphous calcium phosphate (ACP) typically formed at early immersion stages. The further reduction in Ca/P upon Mg decoration suggests partial substitution of Ca^2+^ by Mg^2+^ and Mg-mediated stabilization of ACP, which is known to inhibit premature HAp crystallization [56]. To account for Mg^2+^ substitution, the composite (Ca + Mg)/P ratio was evaluated, yielding values of 0.266 for bare-ZrNTs, 0.59 for Mg-ZrNTs-NLS, and 1.57 for Mg-ZrNTs-WLS. The latter approaches the stoichiometry of apatite at the outermost surface, suggesting the formation of a Mg-substituted ACP/apatite layer that is likely to evolve toward crystalline hydroxyapatite with extended immersion [29,57]. Collectively, these findings indicate that Mg-ZrNTs-WLS exhibits the highest bioactivity after 7 days of SBF soaking, characterized by a thicker Ca-P/Mg overlayer and a divalent cation profile most closely resembling apatite. This composition is expected to enhance interfacial reactivity and promote subsequent bio-osseointegration.

### 3.3. Electrochemical Analysis and Protein Viability

ToF-SIMS surface characterization confirmed Mg presence. To evaluate whether these modifications enhance electrochemical performance, impedance spectroscopy, Tafel analysis, and gas chromatography were performed. Impedance and Tafel methods were used to assess corrosion resistance and biomineralization effects on surface response, both critical indicators of implant viability. Additionally, it was used to identify residual species and confirm chemical purity post-deposition, ensuring that surface-bound contaminants do not interfere with biological integration, as further assessed via protein viability assays.

#### 3.3.1. Impedance Spectroscopy and Tafel Analysis

**Figure 10:**
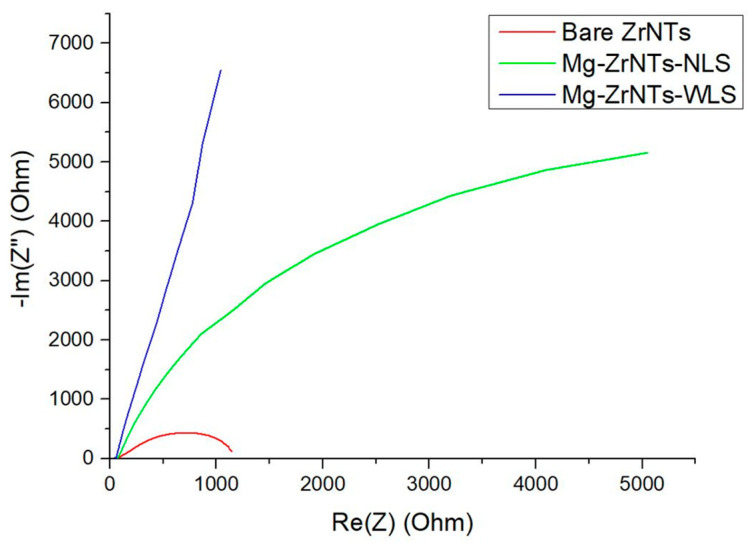
Nyquist plot of samples, bare-ZrNTs, Mg-ZrNTs-NLS, and Mg-ZrNTs-WLS.

These apatite-like coatings prompted further investigation into their resistivity and potential improvements in corrosion resistance. Electrochemical performance was evaluated using impedance spectroscopy and Tafel analysis, with results interpreted in terms of charge-transfer resistance (R_ct_)_,_ interfacial capacitance, and low-frequency film behaviour. This analysis aimed to elucidate the role of Mg decoration and UV-assisted deposition in enhancing the electrochemical properties and bioactive response of bare ZrNTs. The Nyquist plot in Figure 10 clearly differentiates the electrochemical behaviour of bare ZrNTs, Mg-ZrNTs-NLS, and Mg-ZrNTS-WLS. The use of two CPE elements reflects the non-ideal interfacial capacitance and the distributed dielectric behaviour of the porous ZrO_2_ nanotube layer and Mg-modified surface, which cannot be captured by a simplified RC model. The fitted equivalent circuit model (R_1_ + (Q_2_∥R_2_) + (Q_3_∥R_3_)) provides insights into solution resistance (R_1_), charge-transfer resistance (R_ct_ = R_2_), and interfacial capacitance (Q_2_), which are represented in Table 3. Bare-ZNTs nanotubes (red curve) exhibited the highest charge-transfer resistance (R_2_ ≈ 75 Ω), as indicated by the extended arc along the x-axis. This behaviour reflects zirconia’s wide-bandgap ceramic nature, which limits electron exchange across the electrode-electrolyte interface. The constant phase element (Q_2_ = 2.381 × 10^−9^ F·s^(a − 1)) and ideal exponent (α_2_ = 0.9825) confirm a homogeneous and insulating surface, making it highly effective as a corrosion-resistant barrier. The elevated y-axis offset further indicates strong interfacial polarization and minimal ionic mobility, reinforcing its role as a passive protective layer, indirectly confirming the inert nature of the oxide nanotubes.

The nanotube layer functions as an oxide coating while not forming a perfectly dense, inert barrier. Field-grown ZrO_2_ develops a thin, defect-rich barrier layer that appears compact but remains ion-conductive, containing oxygen vacancies and incorporating species such as F^−^. These features allow ionic transport and localized electron tunnelling at the thinned pore bases, which is essential for sustained nanotube growth. In this functional sense, the oxide is not a fully closed, insulating layer despite its attachment to the underlying metal foil. Upon Mg decoration without UV assistance, the Mg-ZrNTs-NLS (green curve), the system showed a modest increase in capacitance (Q_2_ = 4.197 × 10^−9^ F·s^(a − 1)) and a decrease in α_2_ to 0.9678, suggesting increased surface heterogeneity and roughness. However, the charge-transfer resistance remained relatively unchanged (R_2_ ≈ 68.46 Ω), indicating only a marginally better conductivity.

In contrast, the UV-assisted Mg-decorated sample, Mg-ZrNTs-WLS (blue curve), demonstrated a significant reduction in resistance (R_2_ ≈ 55.61 Ω) and a more uniform capacitive response (α_2_ = 0.78), indicating enhanced charge-transfer kinetics. This improvement is attributed to UV-induced oxygen vacancies that facilitate Mg incorporation and promote faster electron exchange [58]. These findings suggest that Mg-ZrNTs-WLS results in greater Mg activity and coverage, enhancing charge exchange and surface responsiveness. Accordingly, this substrate is identified as the most electrochemically active.

The right side of the Nyquist plot reflects high-frequency behaviour and interfacial polarization. Differences are most pronounced between the red curve (bare-ZrNTs) and the blue curve (UV-assisted; Mg-ZrNTs-WLS). The red curve shows a steep, angular tail and the highest y-axis offset, indicating strong interfacial polarization and poor ionic mobility, traits consistent with zirconia’s chemical inertness. The green curve shows a slightly softened tail and reduced offset, suggesting partial relief from polarization effects, though ionic transport remains limited. The blue curve exhibits a flatter high-frequency tail and the lowest y-axis offset, signifying reduced interfacial polarization and improved ion diffusion across the interface. These features are critical for applications requiring dynamic electrochemical interaction. When taken together, the blue sample’s combination of enhanced capacitance, reduced resistance, and improved interfacial behaviour makes it a promising candidate for bioimplant applications, where initial surface activity and moderate conductivity are essential for integration and performance.

**Figure 11:**
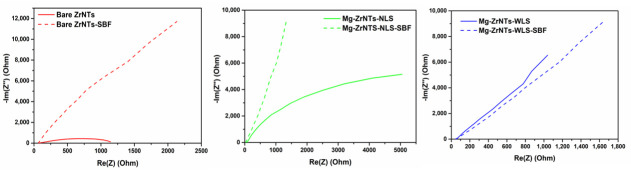
Comparison of Nyquist plots before and after SBF incubation, (**left**) Bare-ZrNTs, (**centre**) Mg-ZrNTs-NLS, and (**right**) Mg-ZrNTs-WLS.

Furthermore, the concept of tuning zirconia’s electrochemical properties via Mg decoration is supported by previous findings, which show that Mg doping reduces the optical bandgap from 5.42 eV to 4.12 eV with increasing Mg content [59]. This doping introduces surface hydroxyl (OH) groups, altering surface chemistry and reactivity. In this work, we exploit these changes to induce apatite-like formation in SBF. Samples were soaked in SBF (pH 7.4) at 37 °C for 7 days to evaluate their biomineralization potential. The resulting Ca/P deposition was reflected in the electrochemical response, with Nyquist curves in Figure 11 showing distinct differences in charge-transfer behaviour and interfacial dynamics across bare and Mg-decorated ZrNTs.

A general reduction in charge-transfer resistance (R_ct_) is observed across all samples, likely indicating the formation of a thin surface layer composed of ionically conductive calcium phosphate (Ca-P) salts. Table 4 summarizes the impedance characteristics of the samples after 7-day immersion in simulated body fluid (SBF, pH 7.4, 37 °C); the fitted parameters are obtained using the equivalent circuit R1 + Q2/R2 + Q3 + C4. Electrochemical responses across the ZrNTs system show distinct trends in both low- and high-frequency regions of the Nyquist plot. On the left side, representing low-frequency capacitive behaviour, the bare ZrNTs (red curve) exhibit the highest capacitance (Q_2_ = 5.425 × 10^−9^ F·s^(a − 1)) and a near-ideal phase exponent (α_2_ = 0.94), consistent with a smooth and stable surface. Despite their insulating nature, these samples show a slight reduction in R_ct_ (R_2_ = 71.24 Ω), suggesting the formation of a tightly bound surface layer that preserves electrochemical integrity [60]. The Mg-ZrNTs-NLS (green curve) shows lower capacitance (Q_2_ = 7.413 × 10^−9^ F·s^(a − 1)) but a more ideal phase exponent (α_2_ = 0.9872), indicating surface smoothing post-SBF. Its R_ct_ drops significantly (R_2_ = 37.54 Ω), implying that SBF exposure may have facilitated the formation of a conductive or ion-permeable layer, enhancing surface activity [61]. The Mg-ZrNTs-WLS (blue curve) maintains high capacitance (Q_2_ = 11.31 × 10^−9^ F·s^(a − 1)) and a consistent phase exponent (α_2_ = 0.9213), with a modest reduction in resistance (R_2_ = 54.5 Ω), indicating sustained electrochemical activity and surface stability. In the high-frequency region, which reflects interfacial polarization, the differences become more pronounced. The red sample retains a steep high-frequency tail and prominent y-axis offset, with Q_3_ = 1.463 × 10^−6^ F·s^(a − 1) and α_3_ = 0.94, confirming its resistive and insulating character. The green sample shows a substantial increase in Q_3_ (3.006 × 10^−6^ F·s^(a − 1)) and a slightly lower α_3_ (0.8909), indicating reduced polarization and improved ion diffusion, likely due to surface restructuring or bioactive layer formation. The Mg-ZrNTs-WLS (blue curve), while slightly less ideal in α_3_ (0.8797), maintains a high Q_3_ (3.148 × 10^−6^ F·s^(a − 1)) and a flatter tail, reflecting efficient ionic transport and minimal polarization. Taken together, the UV-assisted Mg-decorated sample demonstrates the most balanced electrochemical profile post-SBF, combining surface stability with sustained ionic responsiveness. Bulk Mg and Mg-alloy implants are known for their elevated corrosion current densities when exposed to physiological environments. Many recent studies [62,63] on Mg alloys and Mg-based coatings report corrosion current densities in the range of 10^−5^–10^−4^ A/cm^2^ in simulated body fluids, often accompanied by pronounced surface degradation. In contrast, the Mg-decorated ZrNTs developed here exhibit corrosion current densities several orders of magnitude lower, reflecting a fundamentally different corrosion mechanism. In our work, magnesium is not present as a bulk, degradable phase but rather as a surface deposition stabilized as MgO/Mg(OH)_2_ within a chemically robust zirconia nanotube framework. As a result, the electrochemical response is governed primarily by the stable ZrO_2_, while magnesium functions as a surface modifier that influences interfacial charge transfer and promotes controlled calcium phosphate nucleation. Importantly, this surface-level incorporation of Mg does not compromise the structural stability of the underlying nanotube architecture. These traits position it as a strong candidate for bioimplant applications, where initial surface activity, long-term stability, and compatibility with physiological environments are essential for successful osseointegration.

Further assessment on the corrosion response via Tafel plots reinforces the electrochemical trends observed in impedance spectroscopy and can be found in the SI, Figure S3. It demonstrates the formation of a highly protective passive layer [62,64]. Following immersion in simulated body fluid (SBF), all samples exhibited further improvement due to biomineralization effects. These findings underscore the functional role of magnesium as not merely a dopant, but as a bioactive agent that enhances surface conductivity, supports ion exchange, and accelerates the formation of biologically compatible layers. By bridging the gap between corrosion resistance and electrochemical responsiveness, Mg decoration, especially when UV-assisted, offers a pathway toward optimized biointegration, making the Mg-ZrNTs-WLS substrate a compelling candidate for implantable devices that require both initial surface activity and long-term stability in physiological environments [9,26,62].

#### 3.3.2. Protein Viability

To complete the evaluation of surface reactivity and biocompatibility, a protein viability assay was performed using horseradish peroxidase (HRP) and ABTS substrate. This test served as a proxy for assessing whether the Mg-decorated ZrNTs surfaces release any species that compromise protein integrity upon contact. HRP activity was measured after direct loading and exposure to the substrates, with ABTS oxidation used to quantify retained enzymatic function.

**Figure 12:**
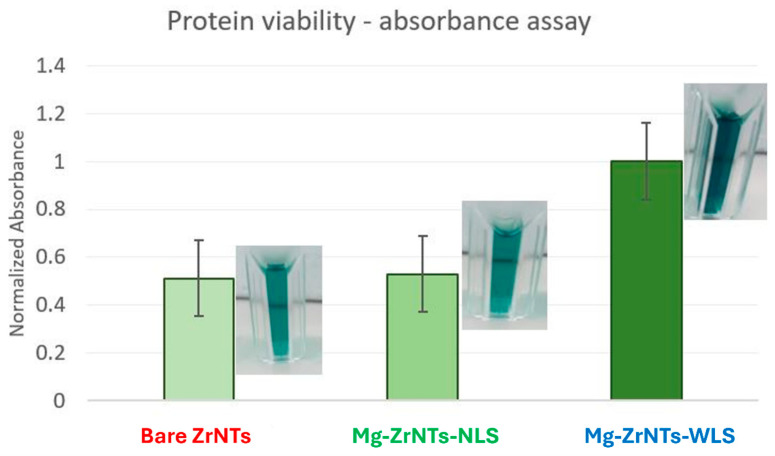
Horseradish peroxidase protein viability using ABTS assay.

The absorbance values obtained from the ABTS colorimetric assay were referenced against the upper detection limit of the instrument, where an absorbance of 2.5 corresponds to the maximum measurable signal (100% absorbance/transmission cutoff). As shown in Figure 12, the Mg-ZrNTs-WLS sample exhibited the highest absorbance intensity at 747 nm, indicating the strongest retention of HRP catalytic activity. In comparison, both bare-ZrNTs and Mg-ZrNTs-NLS samples showed similar absorbance levels, with the NLS variant slightly higher than the unmodified substrate. This hierarchy reflects improved protein immobilization and preserved enzymatic function on Mg-decorated surfaces, particularly when UV light was used during deposition [65].

The enhanced performance of the Mg-ZrNTs-WLS sample is due to the increased surface activation and energy changes after UV exposure and Mg electrodeposition, potentially resulting in the formation of MgOH species on the nanotube surface [42,66]. These changes improve surface wettability and interaction potential with polar amino acid residues, allowing stable adsorption of HRP without compromising its active site [67]. The elevated absorbance observed for Mg-ZrNTs-WLS suggests a higher density of catalytically active, immobilized enzyme. This compatibility is essential for downstream biological processes such as cell adhesion, osteoblast attachment, and osseointegration [68]. The results indicated that HRP remains catalytically active across all samples, confirming that the surfaces, regardless of deposition method, i.e., WLS or NLS, do not induce denaturation or loss of function. These findings suggest that the modified ZrNTs do not release cytotoxic or protein-inactivating species, reinforcing their suitability for biointerface applications where protein stability is essential.

## 4. Highlights and Summary

### 4.1. Electrochemical Deposition Strategy (pLSV ± UV)

The pulsed LSV approach enabled controlled Mg incorporation into the ZrNTs architecture, with UV assistance further modifying the interfacial environment by increasing surface hydroxylation, enhancing hydrophilicity, and promoting a cleaner, more reactive oxide surface during deposition. These combined effects influenced nucleation behaviour, adhesion strength, and the uniformity of the Mg layer.

### 4.2. Morphology and Structural Features

SEM imaging revealed how pLSV parameters influenced Mg distribution and surface texture, while UV-assisted deposition promoted more cohesive and continuous Mg coverage. XRD confirmed the predominantly amorphous nature of the deposited phases, consistent with low-temperature electrochemical growth, and supported the interpretation that interfacial chemistry takes precedence over crystallinity.

### 4.3. Surface Chemistry and Interfacial Bonding (ToF-SIMS and XPS)

ToF-SIMS confirmed Mg incorporation through Mg^+^, MgO^+^, and MgOH^+^ fragments, with UV-assisted deposition shifting the chemistry toward more oxygenated and hydroxylated species, consistent with UV-enhanced surface activation and oxygen uptake. XPS supported this trend, showing progressive Zr 3d and O 1s shifts from bare → NLS → WLS samples, indicating electron withdrawal, increased hydroxylation, and the formation of Zr-O-Mg linkages. Together, these results show that UV not only increases Mg loading but also drives deeper chemical integration and stronger interfacial bonding within the ZrNTs.

### 4.4. Wettability and Interfacial Reactivity (Contact Angle)

Contact angle measurements showed a clear increase in hydrophilicity after Mg deposition, and selective binding of the 8-hydroxyquinoline (8HQ) SAM indicated potential Mg presence rather than a purely physical surface change. ToF-SIMS subsequently validated this by directly detecting Mg-related fragments. The resulting Mg-activated, hydroxylated surface exhibits higher polarity and reactivity, conditions that promote protein interaction, ion uptake, and early CaP nucleation, with UV-assisted samples showing the strongest response.

### 4.5. Electrochemical Stability and Corrosion Behaviour (EIS, Tafel)

Electrochemical measurements showed that Mg deposition increases the surface’s electrochemical activity, reflected in lower initial charge-transfer resistance, with UV-assisted samples exhibiting the strongest response due to their higher degree of Mg oxidation and hydroxylation. This more reactive surface interacts more readily with SBF, accelerating CaP nucleation and leading to a mineral layer that subsequently increases Rct during immersion. Tafel analysis confirmed reduced hydrogen evolution and lower corrosion currents, consistent with the formation of a stabilizing MgO/Mg(OH)_2_ layer that enhances long-term electrochemical robustness.

### 4.6. Bioactivity and Early Mineralization (Protein Assays, ACP Nucleation)

Protein assays showed that Mg-modified surfaces provide a more favourable biochemical environment, with UV-assisted samples supporting the highest protein adhesion and stability due to their increased hydroxylation and surface polarity. This enhanced interfacial chemistry also accelerates early mineralization: the more reactive Mg-rich surface binds Ca^2+^ and PO_4_^3−^ more readily, promoting rapid amorphous calcium phosphate nucleation and growth. Together, these effects demonstrate that Mg, especially when deposited under UV illumination, transforms ZrNTs into a biologically responsive interface capable of initiating early osteogenic processes.

### 4.7. Integrated Perspective

Across all characterization methods, a coherent picture emerges: Mg deposition fundamentally reconfigures the ZrNT interface, and UV assistance amplifies every stage of this transformation. The combined electrochemical–photochemical approach increases surface hydroxylation, strengthens Mg-ZrO_2_ bonding, enhances interfacial conductivity, and accelerates early CaP nucleation, producing a surface that is simultaneously more reactive, more stable, and more biologically responsive. Each technique, structural, chemical, electrochemical, and biological, converges on the same conclusion: UV-assisted pLSV converts inherently bioinert zirconia nanotubes into a chemically integrated, electrochemically robust, and osteoinductively active interface.

## 5. Conclusions

This study demonstrates that pulsed linear sweep voltammetry (pLSV) offers a reliable and scalable route for magnesium (Mg) deposition onto zirconia nanotubes (ZrNTs), with UV-assisted deposition yielding superior interfacial and functional outcomes. Systematic characterization via XRD, SEM, contact angle analysis, ToF-SIMS, XPS, EIS, Tafel polarization, and protein assays consistently confirms that Mg decoration significantly enhances surface wettability, hydroxylation, and chemical stability. UV irradiation during deposition notably promotes stronger Mg adhesion, increased formation of Mg-O and Mg(OH)_2_ species, and robust Mg–ZrO_2_ interfacial bonding, as evidenced by ToF-SIMS. These modifications result in elevated surface polarity, reduced charge-transfer resistance, and facilitated amorphous calcium phosphate nucleation, collectively improving electrochemical robustness and bioactivity. The observed reduction in hydrogen evolution and corrosion current density further supports the formation of a stable passivating MgO/Mg(OH)_2_ layer. Enhanced protein viability underscores the biofunctional potential of the modified surfaces. Altogether, the synergistic effect of electrochemical and UV-assisted Mg deposition transforms ZrNTs into a bioactive and electrochemically resilient interface, offering a promising strategy to overcome the intrinsic bioinertness of zirconia for osteointegrative applications.

## Figures and Tables

**Table 1:** Binding energies of Zr 3d and O 1s peaks, VBMx (*-theoretically), and the V_BB_ values, expressed in units of electron volts [eV] for all samples.

Sample	Zr 3d (in eV)	O 1s (in eV)	VBMx (in eV)	Band Bending V_BB_ (in eV)
**Bare-ZrNTs**	182.12	529.72	5.8 *	-
**Mg-ZrNTs-NLS**	183.0	530.8	-	0.88
**Mg-ZrNTs-WLS**	183.51	531.51	-	+0.51 (1.39)

**Table 2:** XPS surface elemental composition (at%) of samples after being soaked in SBF for 7 days.

Sample	Zr (at%)	O (at%)	C (at%)	Ca (at%)	P (at%)
**Bare-ZrNTs**	2.32	34.23	20.78	8.96	33.71
**Mg-ZrNTs-NLS**	5.55	28.47	15.70	5.36	31.59
**Mg-ZrNTs-WLS**	0.74	15.3	7.25	6.62	29.83

**Table 3:** Impedance characteristics of samples before SBF incubation.

	Bare-ZrNTs	Mg-ZrNTs-NLS	Mg-ZrNTs-WLS
**R1 [Ohm]**	0.1958	0.5261	0.55
**R2 [Ohm]**	75	68.46	55.61
**Q2 F** **·** **s^(a** **− 1)**	2.381 × 10^−9^	4.197 × 10^−9^	94.1 × 10^−9^
**a2**	0.9825	0.9678	0.78
**Q3 F·s^(a − 1)**	27.53 × 10^−6^	4.372 × 10^−6^	3.631 × 10^−6^
**a3**	0.6056	0.8026	0.89
**R3 [Ohm]**	986.1	0.1× 10^6^	10 × 10^3^
**X^2^ (chi-square)**	0.408	0.4946	2.05

**Table 4:** Impedance characteristics of samples after SBF incubation.

Parameter	Bare-ZrNTs	Mg-ZrNTs-NLS	Mg-ZrNTs-WLS
**R1 [Ohm]**	0.014	0.02	0.405
**R2 [Ohm]**	71.24	37.54	54.5
**Q2 F·s^(a − 1)**	5.425 × 10^−9^	7.413 × 10^−9^	11.31 × 10^−9^
**a2**	0.94	0.9872	0.9213
**Q3 F·s^(a − 1)**	1.463 × 10^−6^	3.006 × 10^−6^	3.148 × 10^−6^
**a3**	0.94	0.8909	0.8797
**C4 [F]**	1.2 × 10^−4^	1.04 × 10^−4^	2.5 × 10^−4^
**X^2^ [ohm]**	0.00789	0.0018	0.0022

## Data Availability

The data presented in this study are available only on request from the corresponding author as it is part of an ongoing study.

## Supplementary Materials

The following supporting information can be downloaded at: https://www.mdpi.com/article/10.3390/jfb17030158/s1, Figure S1: SEM images of (a) UV-treated-ZrNTs, and (b) untreated-ZrNTs after 7-day incubation in simulated body fluid (SBF), (c) XRD analysis of Bare ZrNTs, Mg-ZrNTs-NLS, Mg-ZrNTs-WLS, UV-treated ZrNTs, and no UV treatment ZrNTs after SBF incubation; Figure S2: (a) Survey spectra of bare ZrNTs (Red), Mg_ZrNTs_NLS (Green), and Mg_ZrNTs_WLS (Blue), (b) XPS spectra of Zr 4s region for bare ZrNTs (Red), Mg_ZrNTs_NLS (Green), and Mg_ZrNTs_WLS (Blue)—Regions of MgO/MgOH (49.5–51.5 eV range) hidden in the Zr 4s region, highlighted with purple dashed line; Figure S3: Polarization Tafel plots of samples before and after incubation in SBF Table S1: Polarization Tafel plots of samples before and after incubation in SBF.
