# Supplementary material for: Surface-Activated Zirconia Nanotubes with UV-Assisted Mg Deposition: Novel Bioinstructive Implants
Source: J Funct Biomater. 2026 Mar 23;17(3):158. doi: 10.3390/jfb17030158 (PMC13028173; doi:10.3390/jfb17030158)
Supplement: Supplementary file 1 [file jfb-17-00158-s001.zip › jfb-4187943-supplementary.pdf]

SUPPORTING INFORMATION

Surface-Activated Zirconia Nanotubes with UV-Assisted Mg Deposition: Novel Bioinstructive Implants

Swathi N. V. Raghu * , Yomna Badran, Shanmugapriya Periyannan * and Manuela S. Killian

S1: Effects of electrodeposition and UV light exposure on ZrNTs

To investigate how secondary electrochemical treatment influences the fabricated ZrNTs, electrodeposition was first carried out in an Mg-free electrolyte to isolate the effect of the deposition process itself on the nanotube layer. A second specimen underwent the same electrodeposition procedure but with continuous UV illumination during the entire deposition step to assess whether photoactivation further modifies the surface. Following these treatments, all samples were immersed in SBF at 37ºC for 7 days to evaluate their ability to promote CaP nucleation and growth. SEM imaging of the resulting surfaces shows clear differences in CaP coverage and morphology between the untreated, electrodeposited, and UV-assisted electrodeposited ZrNTs, demonstrating the influence of the secondary treatment conditions on subsequent mineralization behaviour.

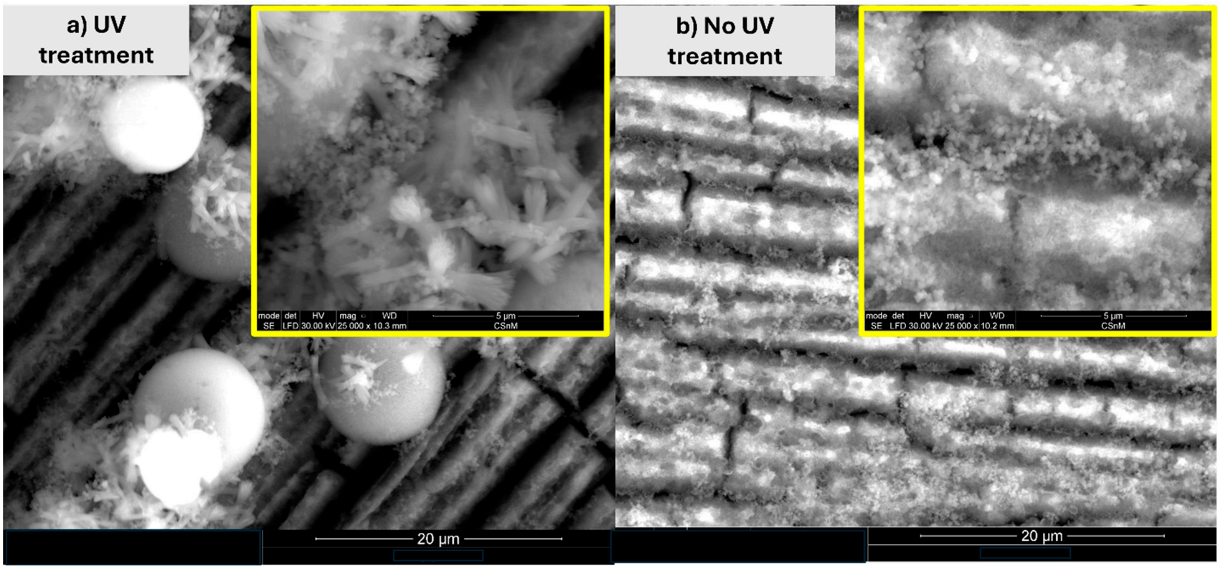

Figure S1: SEM images of a) UV-treated-ZrNTs, and b) untreated-ZrNTs after 7-day incubation in simulated body fluid (SBF)

In the SEM images, panel (a) shows the UV-treated specimen and panel (b) the untreated electrodeposited specimen. The two surfaces exhibit clearly distinguishable CaP morphologies. In (a), the deposits consist predominantly of larger spherical aggregates surrounded by fine, spike-like protrusions, with noticeable spacing between neighbouring features, which are especially noticeable in the inset. In contrast, the surface in (b) displays much smaller spheres

that cluster more tightly together, forming a string of bead-like dispersed layers. Neither specimen shows the characteristic bloom-like or needle-like CaP structures typically associated with fully matured apatite. Overall, UV-treated ZrNTs primarily promote dense particulate nucleation, while electrolyte-treated ZrNTs exhibit only sparse, diffuse deposition, and in comparison, as already discussed in the main manuscript in Figure 3, Mg-ZrNTs-WLS displays high coverage with uniformly fuzzy particle microstructures, which suggests that the combined synergistic effect of Mg and UV assistance governs morphology-directed biomineralization rather than merely increasing nucleation density. A corresponding XRD analysis of these specimens is shown in Figure S1 (c); the XRD patterns obtained after 7-day immersion in simulated body fluid reveal weak apatite-like calcium phosphate (CaP) reflections across all investigated surfaces.

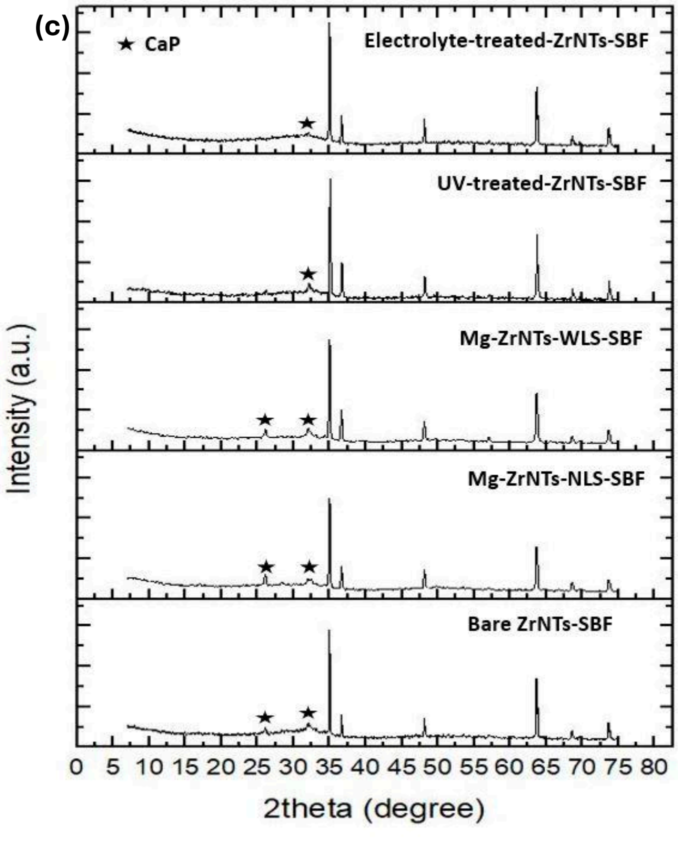

Figure S1: c) XRD analysis of Bare ZrNTs, Mg-ZrNTs-NLS, Mg-ZrNTs-WLS, UV-treated ZrNTs, and no UV treatment ZrNTs after SBF incubation

Electrolyte-treated ZrNTs display a weak and broadened peak, with no noticeable sharpening or intensification of CaP peaks, aligning with the sparse deposition observed in SEM. The UV-treated ZrNTs exhibit slightly more pronounced CaP peak as compared to the untreated samples; however, the persistent peak broadness suggests that UV activation enhances nucleation density

rather than promoting crystallinity. As previously discussed in the main manuscript in Figure 4, among the modified samples, Mg-ZrNTs-WLS maintains moderate but controlled CaP features without strong peak intensification, supporting a morphology-directed growth mechanism rather than bulk crystalline accumulation.

S2: Survey and high-resolution scan of *XPS spectra of Zr 4s for ZrNTs and their Mg-counterparts*

Figure S2 a) displays the survey spectra of ZrNTs and their Mg counterparts. All the spectra were observed to have evidence of only Zr- and O-related binding energies that are relevant to ZrNTs, while, in addition, adventitious carbon was seen as a result of exposure to the ambience. Given that low-level Mg deposition was carried out, its presence was not captured in the survey scans.

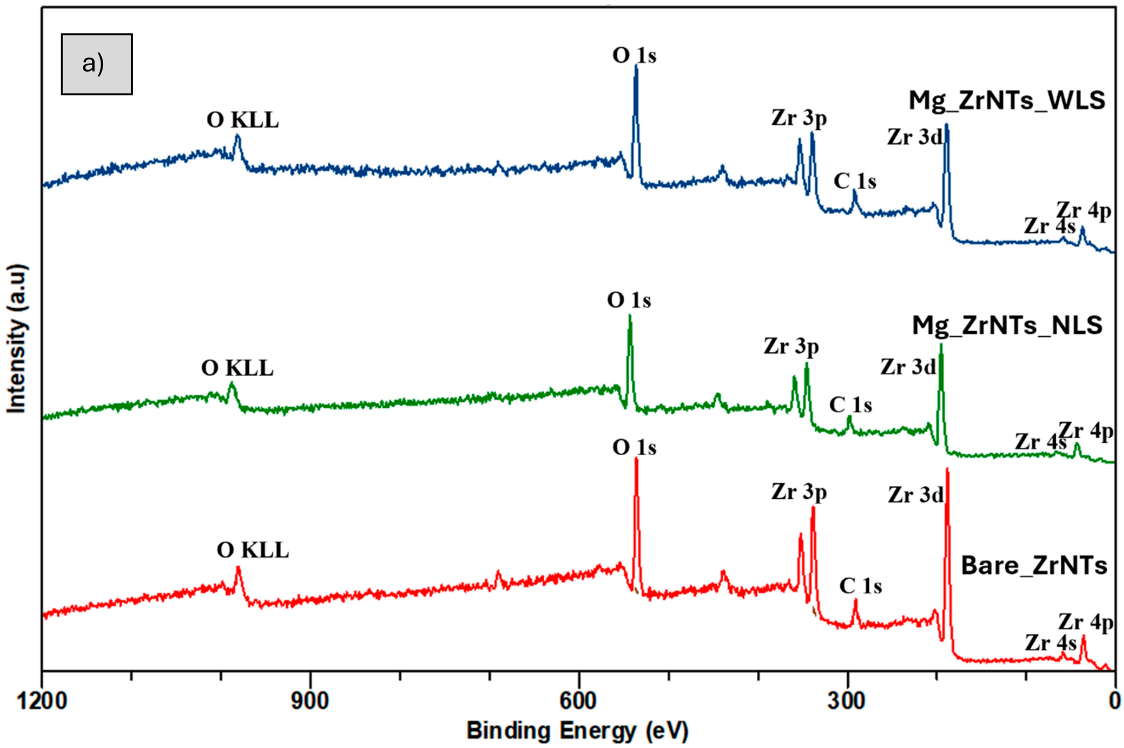

Figure S 2 a) Survey spectra of bare ZrNTs (Red), Mg_ZrNTs_NLS (Green), and Mg_ZrNTs_WLS (Blue).

Figure S2- b) shows the Zr 4s region for ZrNTs and their Mg counterparts. Zr 4s usually occurs in the 48-56 eV range, and that of MgO/$Mg(OH)_2$ occurs in the 49.5-51.5 eV range. In the current material system, Mg is deposited onto ZrNTs, leading to overlapping signals and thus making distinguishing them challenging. Irrespective of the electron withdrawal in Mg_ZrNTs_WLS (and the consequential upward band bending at the surface as confirmed by progressive positive shift in Zr 3d, as was previously discussed in the main manuscript), the shift to lower binding energy

in the Zr 4s peak strongly indicates that a different interaction mechanism is occurring at its interface.

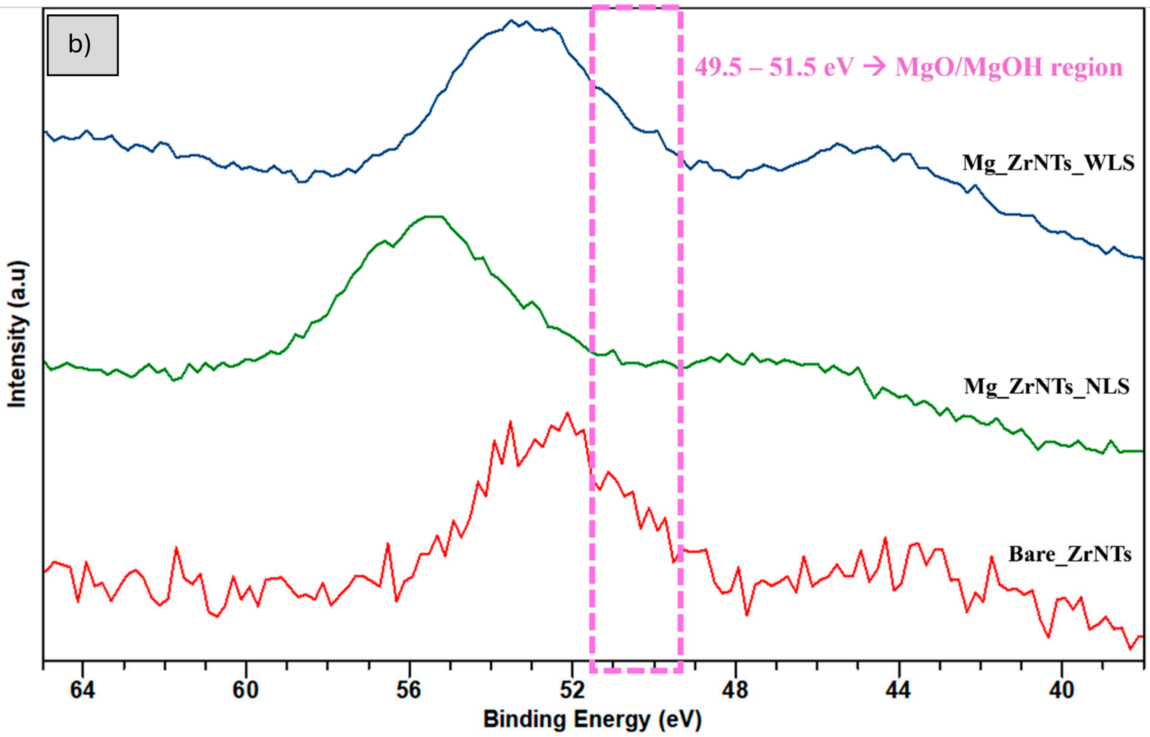

Figure S 2: b) XPS spectra of Zr 4s region for bare ZrNTs (Red), Mg_ZrNTs_NLS (Green), and Mg_ZrNTs_WLS (Blue)—Regions of MgO/MgOH (49.5 - 51.5 eV range) hidden in the Zr 4s region, highlighted with purple dashed line

Under WLS conditions, increased hydroxylation is promoted, hence more polarizable Mg-OH is formed, which means enhanced electron density and better charge redistribution (as well as a better screening effect). Therefore, Zr 4s, which is shallower than Zr 3d, is relatively more sensitive to final-state relaxation and screening effects, exhibiting the difference in shifts between NLS and WLS conditions. Overall, the shifting behaviour in the shallow Zr 4s level of Mg_ZrNTs_WLS reflects a balance between electron withdrawal (which pushes the peak higher) and enhanced final-state screening (which pulls it slightly lower).

S3: Electrochemical behaviour as investigated by *Tafel plots*

In Figure S2, prior to SBF exposure, the bare-ZrNTs (red curve) exhibited the poorest corrosion resistance, with the most negative corrosion potential (Ecorr = -1083 mV), the highest corrosion current density (i_{corr} = 9.8×10^{-3} mA.cm^{-2}), and the lowest polarization resistance (Rp = 7.44×10^{3} Ω.cm^{2}). In Table S1, these values reflect a surface that is highly susceptible to anodic

dissolution and limited in protective capacity. Upon magnesium decoration without UV assistance, the Mg-ZrNTs-NLS (green curve) showed a significant improvement in corrosion resistance: i_{corr} dropped to $2.8x10^{-3}$ $mA.cm^{-2}$ and Rp increased to $2.43x10^{4}$ $\Omega.cm^{2}$, indicating that Mg incorporation reduced the corrosion rate and enhanced surface passivation. The Mg-ZrNTs-WLS specimen (blue curve) showed a dramatic enhancement, with i_{corr} reduced to $1.53x10^{-5}$ $mA.cm^{-2}$ and Rp soaring to 4.01×10^{6} $\Omega.cm^{2}$, which is over two orders of magnitude higher than the bare sample. It demonstrates the formation of a highly protective passive layer. [61] Following immersion in simulated body fluid (SBF), all samples exhibited further improvement due to biomineralization effects.

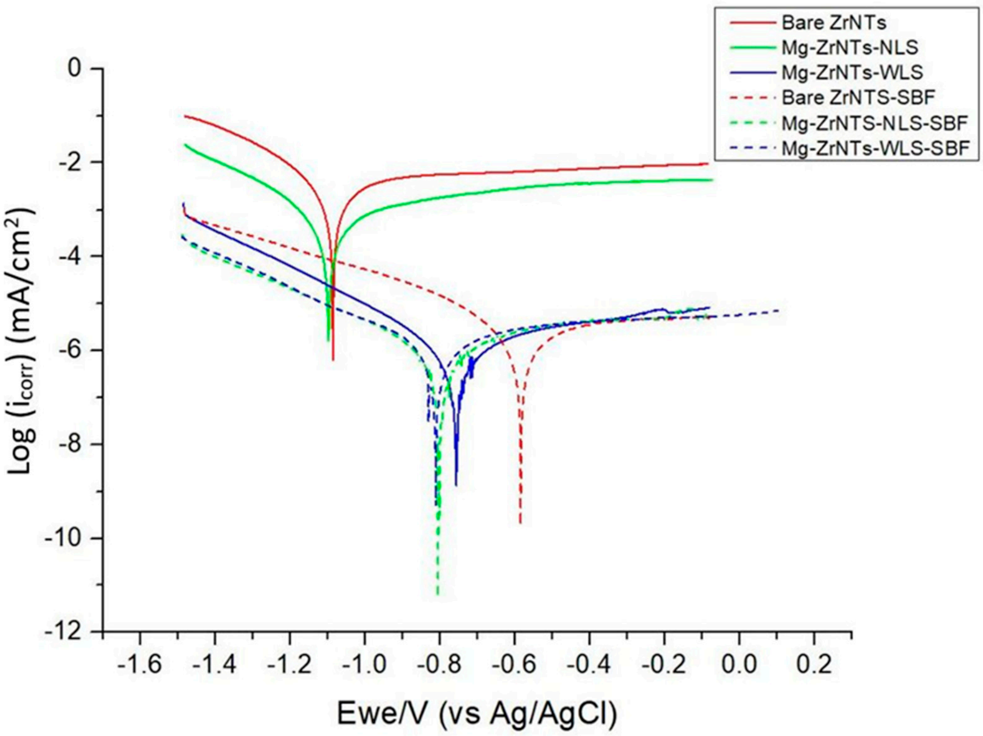

Figure S 3: Polarization Tafel plots of samples before and after incubation in SBF

The bare-ZrNTs showed a marked reduction in i_{corr} ($2.93x10^{-5}$ $mA.cm^{-2}$) and a substantial increase in R_p (2.43×10^{6} $\Omega.cm^{2}$), confirming the deposition of a calcium-phosphate layer that passivates the surface. The Mg-ZrNTs NLS sample achieved the lowest i_{corr} overall ($1.53 x10^{-5}$ $mA.cm^{-2}$) and an R_p of 4.24×10^{6} $\Omega.cm^{2}$, suggesting that Mg sites functioned as nucleation centres for Ca/P precipitation, forming a dense and uniform protective film.

Table S1: Polarization Tafel plots of samples before and after incubation in SBF

Sample	E_{corr} (mV $_{Ag/AgCl}$)	i_{corr} ($mA.cm^{-2}$)	β_a (mV/decade)	β_c (mV/decade)	R_p ($\Omega.cm^2$)
Bare ZrNTs	-1083	$9.8x 10^{-3}$	596.4	233.9	$7.44 x10^{3}$

Mg-ZrNTs-NLS	-1099.175	2.8 x10^{-3}	490.1	231	2.43 x 10^{4}
Mg-ZrNTs-WLS	-758.401	1.53x10^{-5}	365.9	229.9	4.01x10^{6}
Bare ZrNTs-SBF	-583.74	2.93x10^{-5}	504.2	243	2.43x10^{6}
Mg-ZrNTs-NLS-SBF	-801.282	1.53x10^{-5}	360.6	254.8	4.24x10^{6}
Mg-ZrNTs-WLS-SBF	-810.643	2.04x10^{-5}	583.3	356.7	4.72x10^{6}

However, the UV-assisted Mg-decorated sample retained its lead, with an R_p of 4.72×10^6 Ω.cm^2 and a stable i_{corr} of 2.04x10^{-5} mA.cm^{-2}, indicating that the photo-activated surface not only facilitated Mg incorporation but also promoted the formation of the most robust and tightly bound passive layer. The relevance of this improvement becomes clearer when compared against the behaviour of magnesium-based biomaterials. Bulk Mg and Mg-alloy implants are known for their elevated corrosion current densities when exposed to physiological environments. Many recent studies [62][67][68] on Mg alloys and Mg-based coatings report corrosion current densities in the range of 10^{-5}-10^{-4} A/cm^2 in simulated body fluids, often accompanied by pronounced surface degradation. In contrast, the Mg-decorated ZrNTs developed here exhibit corrosion current densities several orders of magnitude lower, reflecting a fundamentally different corrosion mechanism. In our work, magnesium is not present as a bulk, degradable phase but rather as a surface deposition stabilized as MgO/Mg(OH)□ within a chemically robust zirconia nanotube framework. As a result, the electrochemical response is governed primarily by the stable ZrO□, while magnesium functions as a surface modifier that influences interfacial charge transfer and promotes controlled calcium phosphate nucleation. Importantly, this surface-level incorporation of Mg does not compromise the structural stability of the underlying nanotube architecture.
